# Multimodal Evaluation of Surface Topography and Color Stability in Pediatric Restorative Materials Exposed to Antihistamine Syrups

**DOI:** 10.3390/jfb17090462

**Published:** 2026-09-08

**Authors:** Oğuzhan Çağlayan, Nihal Şehkar Oktay, Hüseyin Yüce, Pınar Yılmaz Atalı, Başak Durmuş

**Affiliations:** 1 Department of Pediatric Dentistry, Faculty of Dentistry, Marmara University, Istanbul 34840, Türkiye; basak.altinok@marmara.edu.tr; 2 Department of Basic Medical Sciences, Faculty of Dentistry, Marmara University, Istanbul 34840, Türkiye; nihal.oktay@marmara.edu.tr; 3 Department of Computer Systems, Faculty of Technology, Marmara University, Istanbul 34840, Türkiye; huseyin@marmara.edu.tr; 4 Department of Restorative Dentistry, Faculty of Dentistry, Marmara University, Istanbul 34840, Türkiye; pinar.atali@marmara.edu.tr

**Keywords:** antihistamine syrup, glass hybrid restorative system, nanohybrid composite resin, polyacid-modified composite resin, surface properties

## Abstract

Background/Aim: Restorative materials in pediatric dentistry are frequently exposed to acidic oral liquid medications, which may alter their surface properties and color. This in vitro study compared the surface roughness, three-dimensional topography, and color stability (ΔE_00_) of a glass hybrid restorative system (GH), a polyacid-modified composite resin (PMC), and a nanohybrid bulk-fill resin composite (NHC) following a standardized repeated-exposure protocol to two pediatric antihistamine syrups: ketotifen (Zaditen^®^) and cetirizine (Zyrtec^®^). Materials and Methods: Disc-shaped specimens were randomly allocated to three immersion subgroups per material (Zaditen^®^, Zyrtec^®^, distilled water). After baseline measurements (t0), specimens underwent 5000 thermal cycles (t1), then immersion in the assigned solution for 5 min, twice daily, for 6 days (t2). Ra and ΔE_00_ were evaluated quantitatively; surface degradation was further characterized descriptively using 3D optical profilometry and SEM. Data were analyzed using a three-way mixed-design ANOVA (Time × Material × Solution). Results: Antihistamine syrup exposure was associated with significant changes in surface topography and color stability (*p* < 0.001), with large-to-moderate effect sizes for time and its interactions. GH showed the highest Ra and largest color change, but much of this occurred after thermocycling, before syrup exposure. NHC and PMC generally showed smaller changes than GH. 3D profilometry and SEM revealed material-specific morphologies, interpreted descriptively rather than as confirmed degradation mechanisms. Conclusion: Standardized repeated exposure to ketotifen and cetirizine syrups was associated with material-dependent changes in surface topography and color stability, with GH showing the greatest change and NHC/PMC comparatively greater stability; clinical extrapolation requires further study.

## 1. Introduction

Restorative materials used in pediatric dentistry are continuously required to withstand a wide range of biological, chemical, and mechanical challenges inherent to the dynamic oral environment [1,2]. Preserving the structural integrity and aesthetic properties of these materials is critical for maintaining masticatory function and ensuring the long-term oral health of children [3]. However, exogenous factors, particularly dietary acids and the frequent administration of pediatric oral liquid medications (POLMs), consistently pose a severe acidic and cariogenic threat to these restorations [4].

Recent evidence indicates that both dietary liquids and therapeutic formulations, such as iron supplements and antacid syrups, can degrade the organic matrix of composite resins, adversely affecting surface roughness and color stability [5,6,7]. Among pediatric medications, antihistamine syrups (e.g., those containing ketotifen and cetirizine) are frequently prescribed for the long-term management of chronic conditions, including allergic rhinitis, atopic dermatitis, and asthma, resulting in prolonged contact with dental surfaces [7]. Because the endogenous pH of most pediatric syrups falls below the critical demineralization threshold of 5.5, chronic use of these formulations has been shown to induce a drop in biofilm pH comparable to that of sucrose [8]. Chronic acidic exposure raises the surface roughness of restorative materials, which in turn accelerates aesthetically compromising discoloration. Clinically, an increase in surface roughness above the critical threshold of 0.20 µm promotes bacterial adhesion, biofilm formation, and secondary caries, while compromised color stability often necessitates the premature replacement of restorations [9,10,11].

To counter these challenges, a variety of materials are preferred in pediatric restorative dentistry, including glass hybrid restorative systems (GH), polyacid-modified composite resins (PMC), and nanohybrid composite resins (NHC). Glass hybrid systems provide chemical adhesion to dental hard tissues and fluoride release, whereas PMCs combine the benefits of glass ionomers with the improved durability of resin-based materials. Nanohybrid composites, on the other hand, offer superior mechanical strength, high filler loading, and excellent polishability [9,10]. Because these materials differ significantly in their resin matrices, filler sizes, and polymerization mechanisms, their susceptibility to hydrolytic degradation and acidic attacks from liquid medications varies considerably.

While several studies have investigated the general surface roughness and microhardness of primary teeth and basic restorations after exposure to various liquid medications [12], the precise three-dimensional (3D) surface topographical changes and color stability of newer restorative material classes—specifically contemporary glass hybrid restorative systems (e.g., Equia Forte HT) and nanohybrid bulk-fill composites—under a standardized repeated pediatric antihistamine-exposure protocol remain largely uncharacterized. In particular, whether the protective nano-filled resin coat of glass hybrids provides long-term chemical durability compared to polished resin-based materials under such a challenge is currently unknown. Furthermore, where literature on pediatric syrup exposure does exist, the exact topographic degradation mechanisms often remain unclear because conventional two-dimensional (2D) linear roughness evaluations (Ra) provide limited qualitative information regarding the true nature of surface wear [13]. To overcome this limitation, 3D optical profilometry, which encompasses volumetric and spatial parameters such as maximum peak-to-valley height (Sz) and kurtosis (Sku), is essential for distinguishing between complex degradation patterns, such as the hydrothermal delamination of protective coatings versus the selective chemical erosion of the resin matrix [14]. The present study addresses this dual gap by combining 2D linear roughness, 3D non-contact optical profilometry, and SEM imaging to differentiate between distinct micro-morphological wear patterns, such as coating delamination versus selective matrix dissolution, under a simulated chronic antihistamine challenge.

Therefore, the aim of this in vitro study was to evaluate and compare the effects of two commonly prescribed antihistamine syrups (Zaditen^®^ and Zyrtec^®^) on the surface roughness, 3D surface topography, and color stability (ΔE) of GH, PMC, and NHC restorative materials. A multi-analytical approach incorporating contact profilometry, non-contact 3D optical profilometry, scanning electron microscopy (SEM), and spectrophotometric analysis was employed to deeply assess the distinct degradation patterns. The null hypotheses tested in this study were that there would be no statistically significant differences in surface roughness (Ra) or color stability (ΔE) among the three restorative materials (material effect); exposure to different immersion media (Zaditen^®^, Zyrtec^®^, and distilled water) would not significantly alter surface roughness or color stability (solution effect); and there would be no significant differences in surface and color parameters across the evaluated time intervals (time effect).

## 2. Materials and Methods

### 2.1. Study Design and Sample Size

Sample size was estimated using G*Power 3.1 for a three-way mixed-design ANOVA. An effect size of f = 0.40 was derived from the between-group Ra means and pooled standard deviation reported by Yıldız and Ataş [15] for compomer–GIC–composite comparisons under pediatric drug exposure, corresponding to a large partial η^2^ of approximately 0.14. With α = 0.05 and 1 − β = 0.80, the minimum required *n* per subgroup was 9. To account for potential specimen loss or measurement error, *n* = 10 was used and randomly allocated (*n* = 10 each, using a computer-generated random number sequence in Microsoft Excel to ensure unbiased allocation) to three immersion subgroups per material.

### 2.2. Syrup Characterization

The pH of each syrup was measured in triplicate at 25 °C using a calibrated pH meter (FiveEasy FE20, Mettler-Toledo AG, Greifensee, Switzerland). Mean pH values were 5.07 for Zaditen^®^ and 4.75 for Zyrtec^®^ (Table 1).

### 2.3. Specimen Preparation

Three restorative materials were evaluated: a glass hybrid restorative system (Equia Forte HT Fil; GC America, Alsip, IL, USA), a polyacid-modified composite resin (Dyract XP; Dentsply Sirona, Konstanz, Germany), and a nanohybrid bulk-fill resin composite (Kulzer Bulk One; Kulzer, Hanau, Germany) (Table 2). All specimens were prepared by a single operator under standardized conditions using cylindrical silicone molds with an internal diameter of 8 mm and a height of 2 mm. Molds were placed on a glass slab covered with a Mylar strip. For light-polymerization, an LED curing unit (Elipar S10, 3M ESPE, St. Paul, MN, USA) with a manufacturer-specified irradiance of 1200 mW/cm^2^ was utilized. The irradiance was verified using a digital radiometer prior to the initiation of the study. During polymerization, the active tip of the curing unit was held in direct contact (0 mm distance) with the overlying Mylar strip to standardize the light distance. Based on these parameters, the calculated total radiant exposure delivered to each specimen was 24 J/cm^2^ (1.2 W/cm^2^ × 20 s).

For GH: Capsules were mixed for 10 s in an automatic mixer (Dentomat Compact, Dentsply Sirona) and dispensed into the molds. After a 2.5 min initial setting time, specimens were removed, and excess material was eliminated using a fine-grit bur. A uniform layer of a nano-filled resin coating (Equia Forte Coat) was applied and light-cured for 20 s using an LED curing unit (Elipar S10, 3M ESPE, St. Paul, MN, USA; 1200 mW/cm^2^). No mechanical polishing was performed, according to the manufacturer’s instructions.

For PMC and NHC: Materials were injected directly into the molds. A Mylar strip and a glass slide were placed on top to extrude excess material and ensure a flat surface. Specimens were light-cured for 20 s on the top surface, inverted, and cured for an additional 20 s on the bottom. Following polymerization, the measurement surfaces of the PMC and NHC specimens were polished using a four-step aluminum oxide disc system (OptiDisc, Kerr, Orange, CA, USA) under water cooling.

Surface preparation followed the manufacturer-recommended clinical protocol for each restorative system. Accordingly, the GH specimens received the proprietary resin coating without subsequent mechanical polishing, whereas the PMC and NHC specimens underwent standardized mechanical polishing. Therefore, the resulting baseline surfaces reflected the material-specific clinical finishing protocols rather than an identical surface-treatment procedure across all three materials.

### 2.4. Thermocycling

All specimens underwent 5000 thermal cycles between 5 °C and 55 °C, with a 30 s dwell time and a 10 s transfer time (Thermocycler, SD Mechatronik GmbH, Feldkirchen-Westerham, Germany), simulating approximately 6 months of clinical service based on an estimated 10,000 cycles per year [16].

### 2.5. Immersion Protocol

Following thermocycling, specimens were subjected to a standardized repeated medication-exposure protocol. Specimens were immersed in 10 mL of the assigned solution for 5 min, twice daily, for 6 consecutive days. The immersion procedure was adapted from the antihistamine-exposure model described by Costa et al. [17] and was designed to provide standardized and reproducible exposure conditions for comparison among the tested restorative materials. The medication-immersion protocol was considered a chemical exposure challenge distinct from the preceding thermocycling procedure and was not intended to represent a specific duration of clinical exposure. Between immersions, specimens were rinsed with distilled water for 10 s and stored in artificial saliva (1.5 mmol/L CaCl_2_, 50 mmol/L KCl, 0.9 mmol/L KH_2_PO_4_, 20 mmol/L Tris buffer, pH 7.4) at 37 °C, as described previously [17]. The artificial saliva was refreshed every 24 h. As this standardized protocol involved uninterrupted medication contact, it should be regarded as an accelerated in vitro chemical challenge rather than a direct simulation of the dynamic intraoral exposure, in which the medication is swallowed and subsequently diluted and cleared by saliva.

### 2.6. Surface Roughness and 3D Topography

Linear surface roughness (Ra) was measured by contact profilometry (Surftest SJ-211, Mitutoyo, Kawasaki, Japan) using a 0.8 mm cut-off, 4.0 mm evaluation length, and three parallel traces per specimen intersecting the geometric center; the arithmetic mean of the three traces was recorded. Areal roughness parameters (Sa, Sz, Sp, Sv, Sku) were computed in accordance with ISO 25178-2 [18]. using a non-contact 3D optical profilometer (S lynx, Sensofar Metrology, Terrassa, Spain), with a 20× objective and a 2.5 × 2.5 mm^2^ measurement field acquired at the geometric center of each specimen. Ra and areal parameters were therefore obtained from measurement fields of different scales and are not directly comparable in absolute magnitude.

For contextual interpretation, Ra values were considered in relation to the previously established 0.20 µm plaque-retention reference value [9]; however, this value was used only as a contextual reference rather than a validated clinical acceptability threshold for the tested materials, and statistical significance and this contextual comparison were evaluated separately.

### 2.7. Color Stability Measurements

For the clinical interpretation of color change, ΔE_00_ = 1.80 was prespecified as the single primary acceptability threshold. Accordingly, values of ΔE_00_ ≤ 1.80 were considered clinically acceptable, whereas values exceeding 1.80 were considered potentially clinically unacceptable. Statistical significance and clinical acceptability were evaluated separately.

$$\Delta E_{00}=\sqrt{{\left(\frac{\Delta L^{\prime }}{k_{L}S_{L}}\right)}^{2}+{\left(\frac{\Delta C^{\prime }}{k_{C}S_{C}}\right)}^{2}+{\left(\frac{\Delta H^{\prime }}{k_{H}S_{H}}\right)}^{2}+R_{T}\left(\frac{\Delta C^{\prime }}{k_{C}S_{C}}\right)\left(\frac{\Delta H^{\prime }}{k_{H}S_{H}}\right)}$$

Measurements were recorded at three intervals:

ΔE_1_: Color change between baseline (t0) and post-thermocycling (t1).

ΔE_2_: Color change between post-thermocycling (t1) and post-immersion (t2).

ΔE_3_: Overall color change between baseline (t0) and post-immersion (t2).

### 2.8. Scanning Electron Microscopy (SEM)

Twelve representative specimens (controls and syrup-exposed specimens from each material) were selected for micro-morphological examination. Because of their non-conductive nature, specimens were sputter-coated with a gold–palladium alloy at 18 mA for 180 s (SC7620, Quorum Technologies, Laughton, UK). Surface morphology was then examined using a scanning electron microscopy (EVO MA10, Carl Zeiss AG, Oberkochen, Germany) at an accelerating voltage of 10 kV. Images were captured at ×500, ×1000, ×5000, and ×10,000 to detect matrix degradation, filler exposure, and coating delamination.

Before the experimental procedures and measurements were initiated, specimens for SEM and 3D optical profilometry were randomly selected from each experimental subgroup and their assignments were recorded. Thus, specimen selection was completed before any surface roughness or morphological data were available and was not based on roughness values, the magnitude of surface alterations, or specific morphological characteristics. These predefined assignments were maintained throughout the study to minimize the risk of post hoc selection bias.

### 2.9. Statistical Analysis

Statistical analyses were performed using IBM SPSS Statistics v27.0 (IBM Corp., Armonk, NY, USA). Descriptive statistics (mean, standard deviation, minimum, maximum) were calculated. Normality was verified using the Shapiro–Wilk test and variance homogeneity using Levene’s test. A three-way mixed-design ANOVA (Time as within-subject factor; Material and Solution as between-subject factors) was applied to Ra and ΔE_00_ data to evaluate the effects of time, restorative material, immersion solution, and their interactions. Mauchly’s test was used to evaluate the assumption of sphericity for the within-subject factor (time); where sphericity was not violated, sphericity-assumed F values are reported. Bonferroni-adjusted post hoc tests were used for pairwise comparisons. Partial eta-squared (η^2^p) with 95% confidence intervals was calculated to indicate the magnitude of the main effects and interactions. The level of statistical significance was set at *p* < 0.05.

## 3. Results

### 3.1. Surface Roughness (Ra)

The mean surface roughness (Ra) values and standard deviations of the tested restorative materials at different measurement intervals are presented in Table 3. Repeated-measures ANOVA revealed that surface roughness was significantly influenced by time, material type, immersion solution, and the interaction of these factors (*p* < 0.001).

For NHC, no statistically significant differences were observed among Ra_t0, Ra_t1, and Ra_t2 in the distilled-water control group (Ra_t2 = 0.28 ± 0.01 µm; *p* > 0.05). Statistically significant increases in surface roughness were observed following exposure to both antihistamine syrups. In the Zaditen^®^ group, Ra_t2 (0.29 ± 0.04 µm) was significantly higher than Ra_t0 (0.23 ± 0.04 µm) and Ra_t1 (0.23 ± 0.03 µm) (*p* = 0.022 and *p* = 0.005, respectively). A similar pattern was observed in the Zyrtec^®^ group, in which Ra_t2 (0.31 ± 0.04 µm) was significantly higher than both Ra_t0 and Ra_t1 (*p* = 0.003 and *p* = 0.005, respectively).

The GH showed the highest absolute Ra values among the tested materials. In the GH control group, Ra_t2 (0.84 ± 0.16 µm) was significantly higher than Ra_t0 (*p* < 0.001). Similarly, in the Zaditen^®^ and Zyrtec^®^ groups, Ra_t2 values (1.01 ± 0.10 µm and 0.96 ± 0.10 µm, respectively) were significantly higher than their corresponding baseline values (both *p* < 0.001). However, these Ra_t0–Ra_t2 comparisons represent the cumulative changes occurring after thermocycling and subsequent solution exposure and should not be interpreted as effects of antihistamine exposure alone.

Significant increases in Ra were also observed for PMC. In the distilled-water control group, Ra_t2 (0.42 ± 0.13 µm) was significantly higher than Ra_t0 (*p* = 0.048). In the Zaditen^®^ group, Ra_t2 (0.41 ± 0.10 µm) was significantly higher than both Ra_t0 and Ra_t1 (*p* = 0.016 and *p* = 0.011, respectively). In the Zyrtec^®^ group, Ra_t2 (0.42 ± 0.06 µm) was also significantly higher than both earlier measurement stages.

### 3.2. 3D Optical Profilometry and Scanning Electron Microscopy

**Figure 1:**
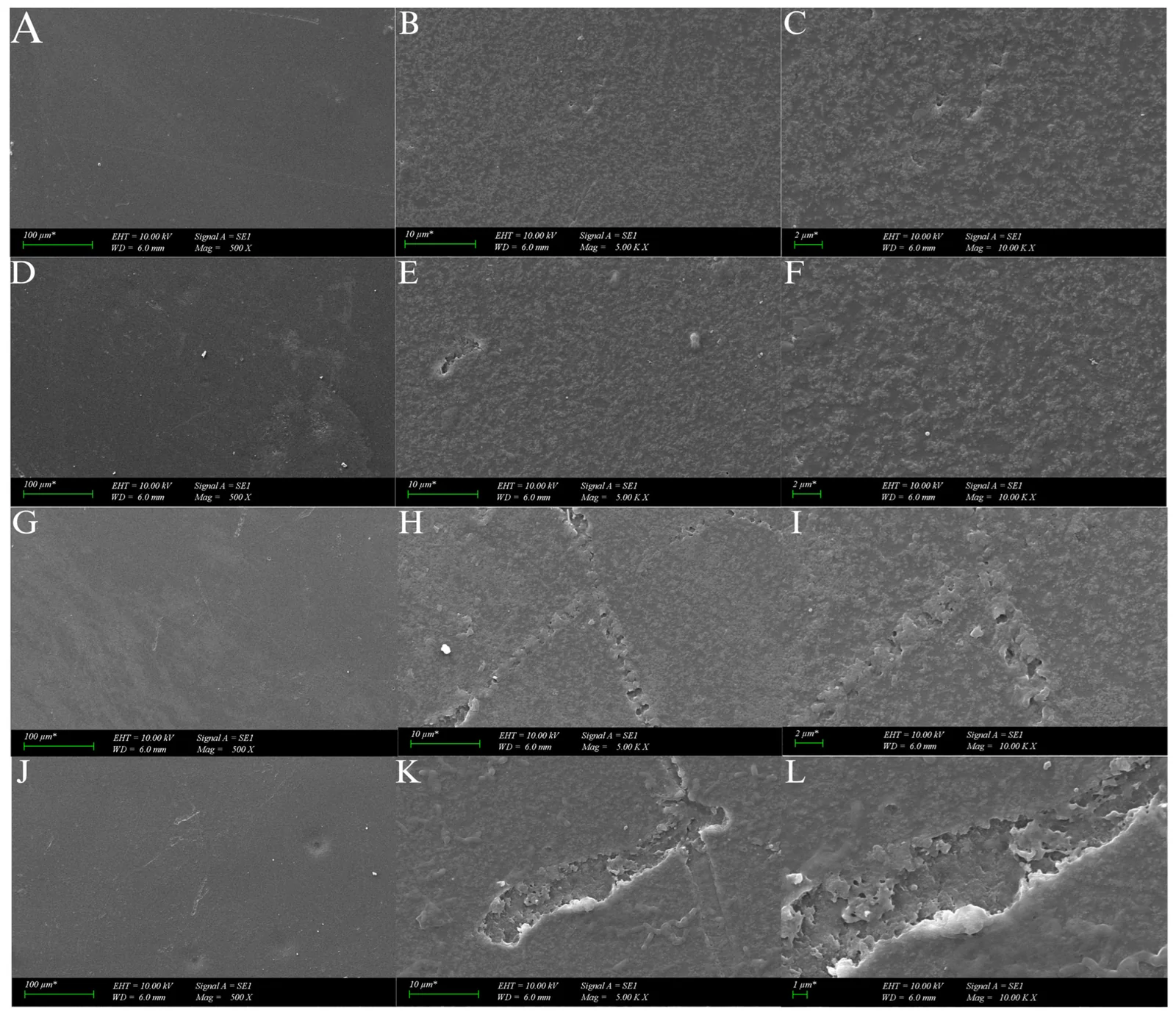
Representative SEM images of the GH specimens: (**A**–**C**) at baseline; (**D**–**F**) after thermocycling; (**G**–**I**) after thermocycling and subsequent Zaditen^®^ immersion; and (**J**–**L**) after thermocycling and subsequent Zyrtec^®^ immersion (×500, ×5000, and ×10,000, respectively). Localized layer separation and interfacial microgaps were observed, potentially indicating disruption of the nano-filled resin coating. EHT: electron high tension (accelerating voltage); WD: working distance; SE1: secondary-electron detector; Mag: magnification.

**Figure 2:**
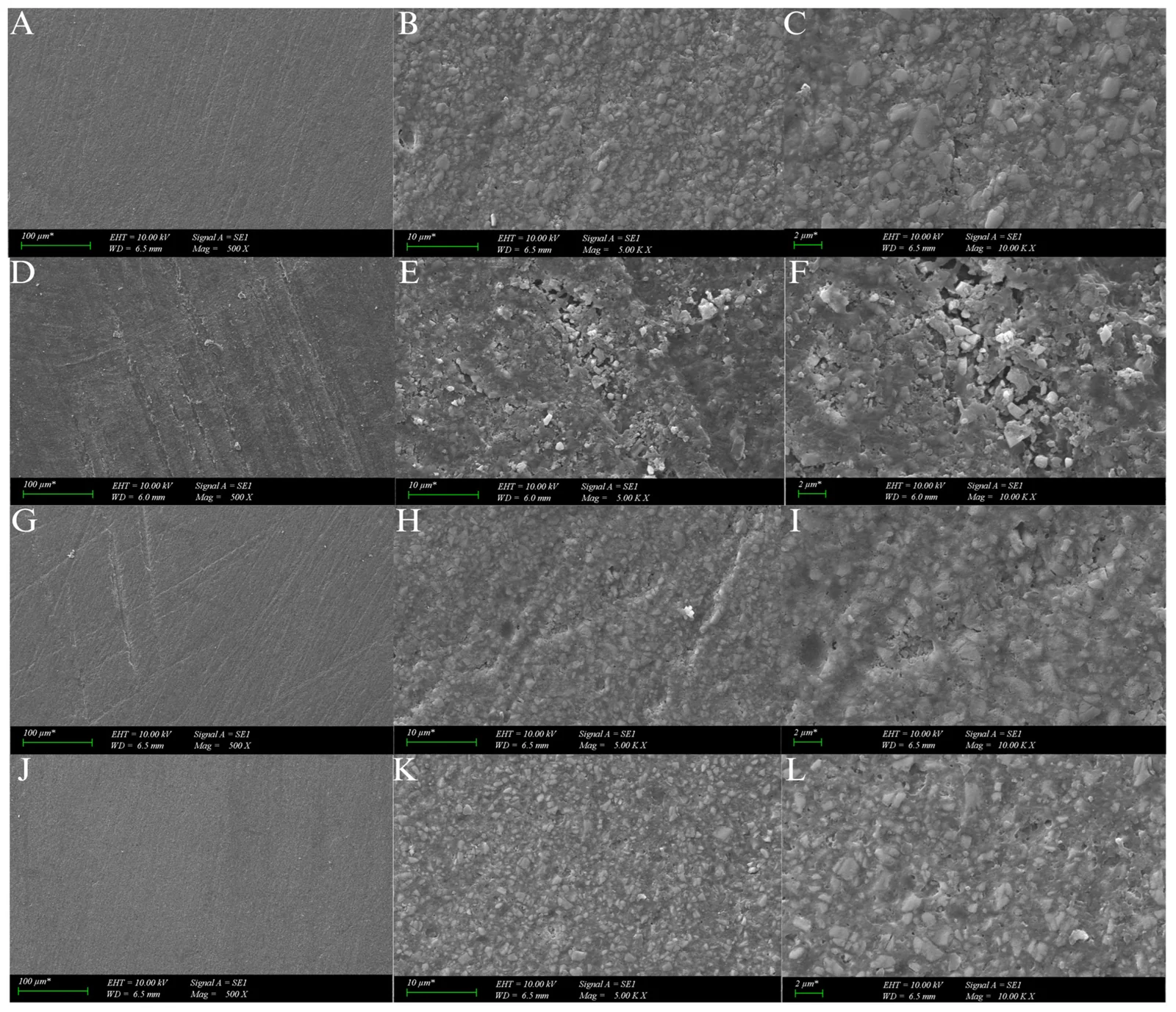
Representative SEM images of the PMC specimens: (**A**–**C**) at baseline; (**D**–**F**) after thermocycling; (**G**–**I**) after thermocycling and subsequent Zaditen^®^ immersion; and (**J**–**L**) after thermocycling and subsequent Zyrtec^®^ immersion (×500, ×5000, and ×10,000, respectively). Localized layer separation and interfacial microgaps were observed, potentially indicating disruption of the nano-filled resin coating. EHT: electron high tension (accelerating voltage); WD: working distance; SE1: secondary-electron detector; Mag: magnification.

**Figure 3:**
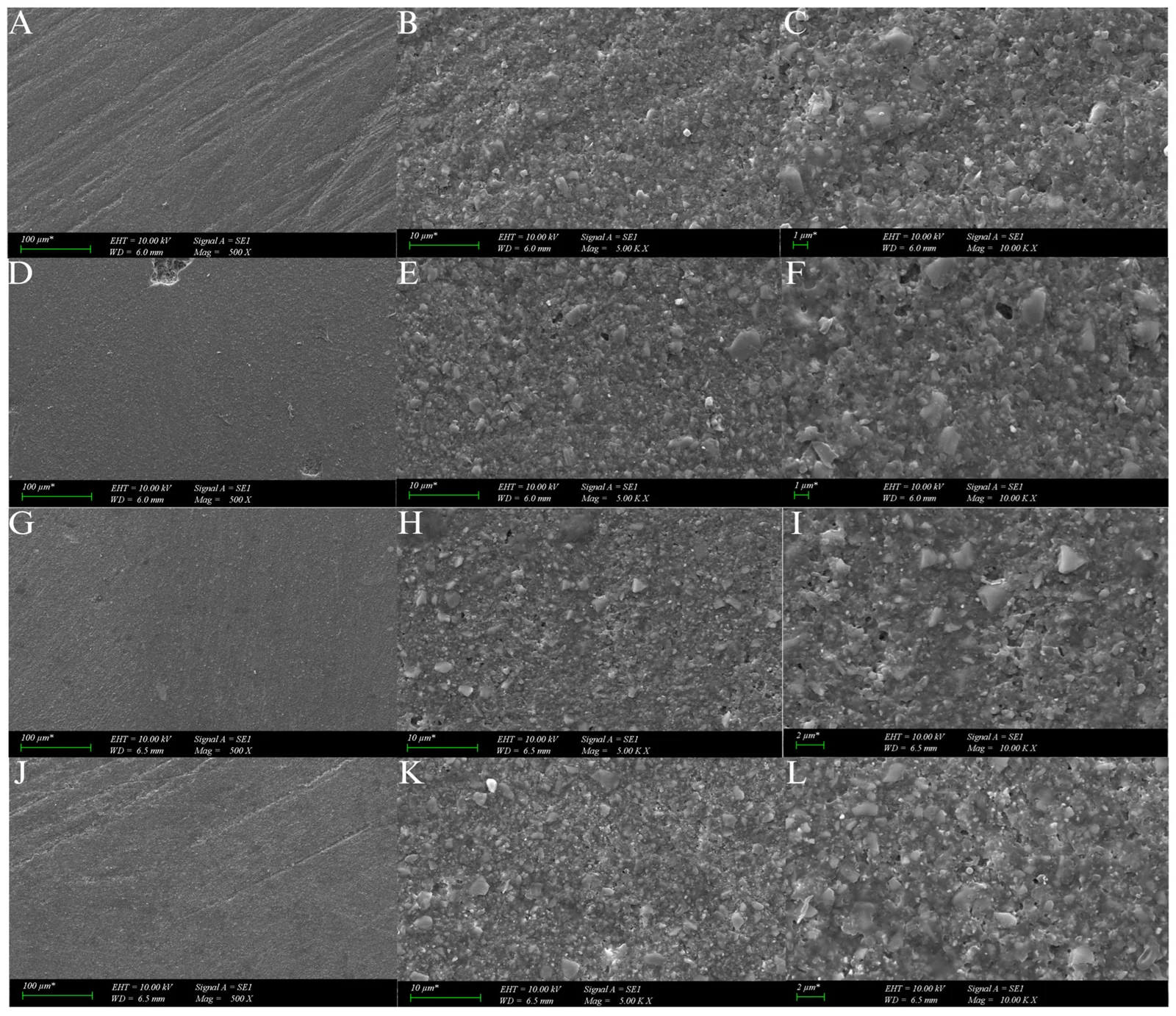
Representative SEM images of the NHC specimens: (**A**–**C**) at baseline; (**D**–**F**) after thermocycling; (**G**–**I**) after thermocycling and subsequent Zaditen^®^ immersion; and (**J**–**L**) after thermocycling and subsequent Zyrtec^®^ immersion (×500, ×5000, and ×10,000, respectively). Localized layer separation and interfacial microgaps were observed, potentially indicating disruption of the nano-filled resin coating. EHT: electron high tension (accelerating voltage); WD: working distance; SE1: secondary-electron detector; Mag: magnification.

**Figure 4:**
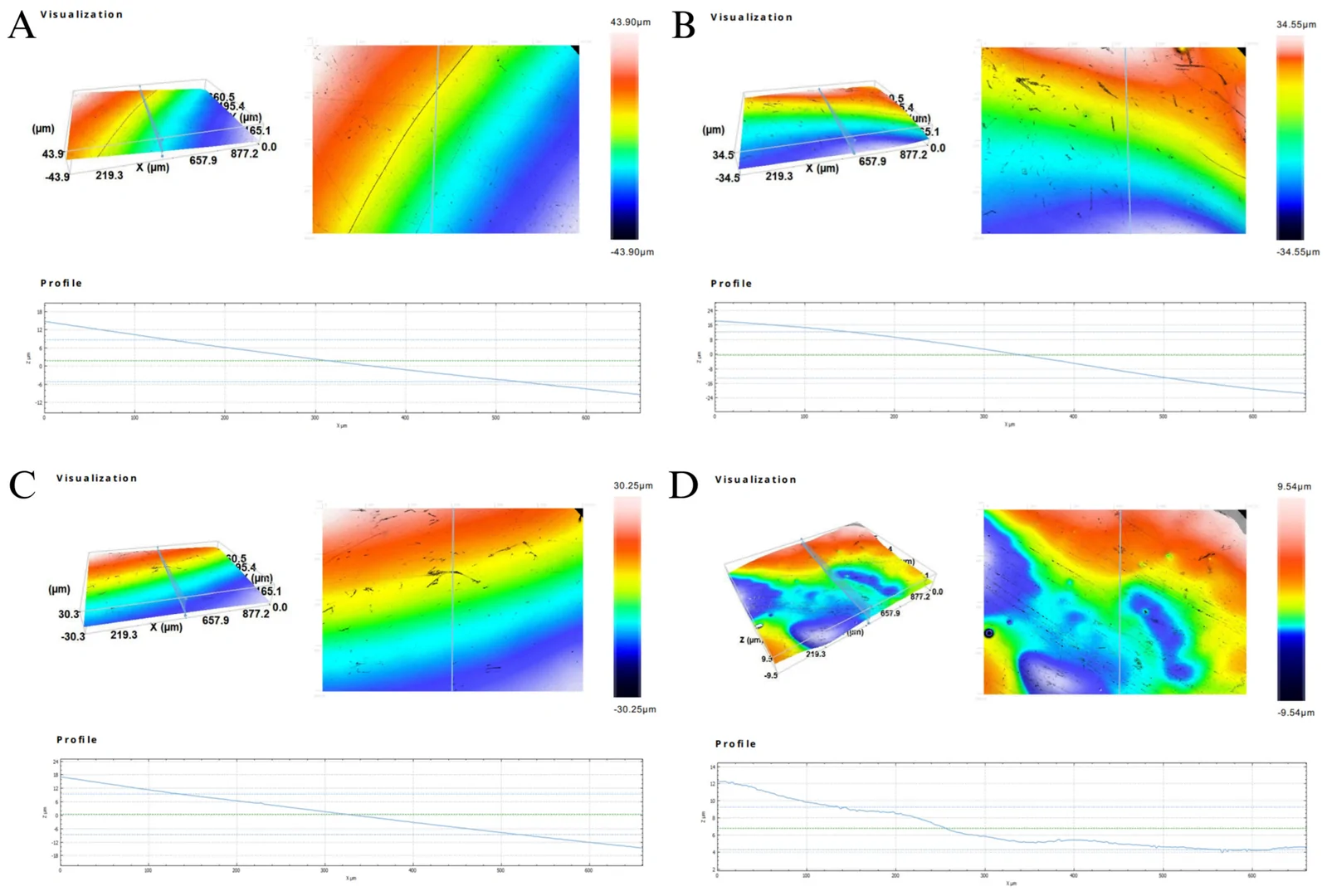
3D optical profilometry images of the GH group: (**A**) control; (**B**) after thermocycling; (**C**) after Zaditen^®^ immersion; (**D**) after Zyrtec^®^ immersion. Profile graph represents the surface-height variation (Z, µm) along this measurement path (X, µm).

**Figure 5:**
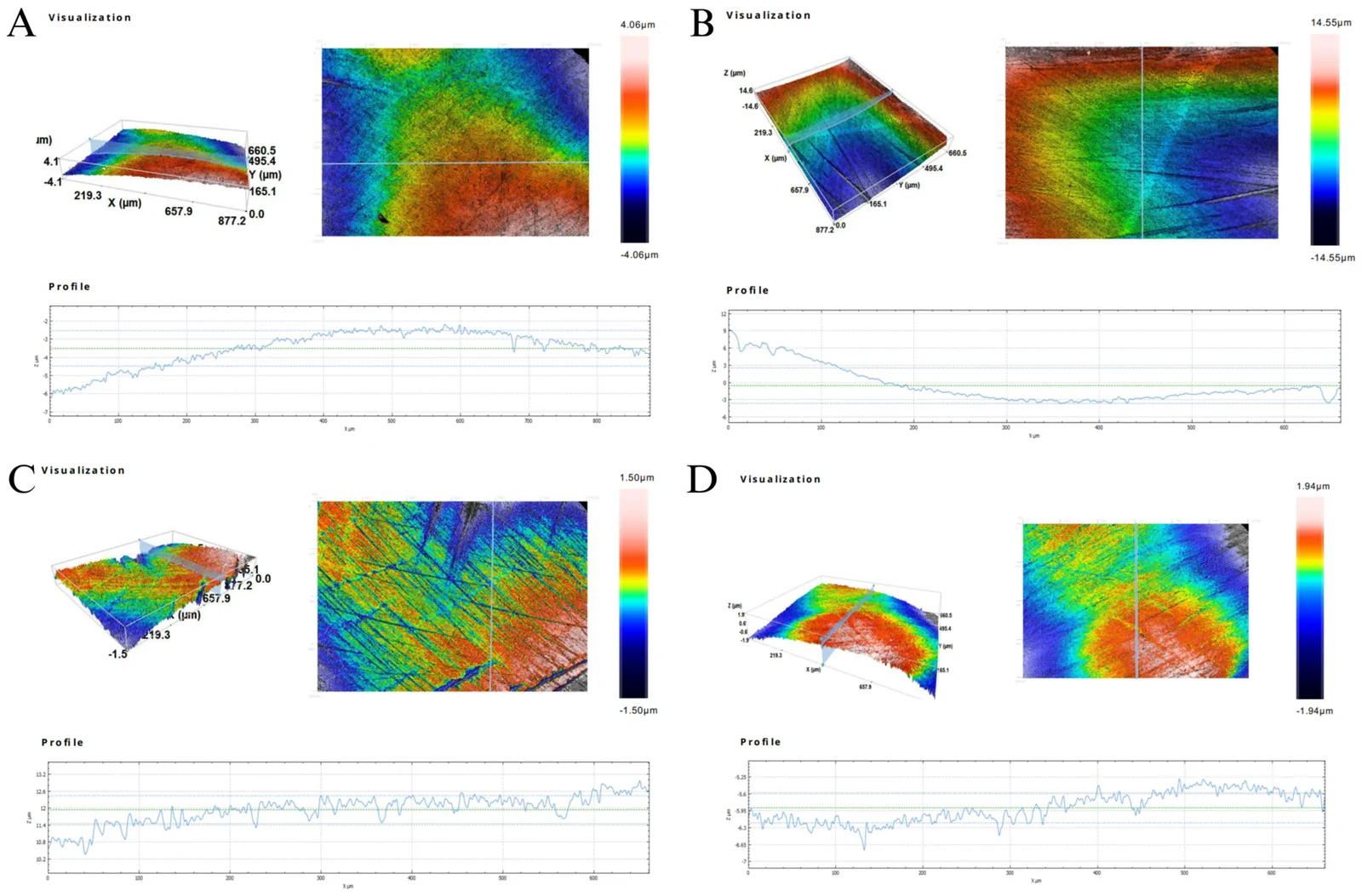
3D optical profilometry images of the PMC group: (**A**) control; (**B**) after thermocycling; (**C**) after Zaditen^®^ immersion; (**D**) after Zyrtec^®^ immersion. Profile graph represents the surface-height variation (Z, µm) along this measurement path (X, µm).

**Figure 6:**
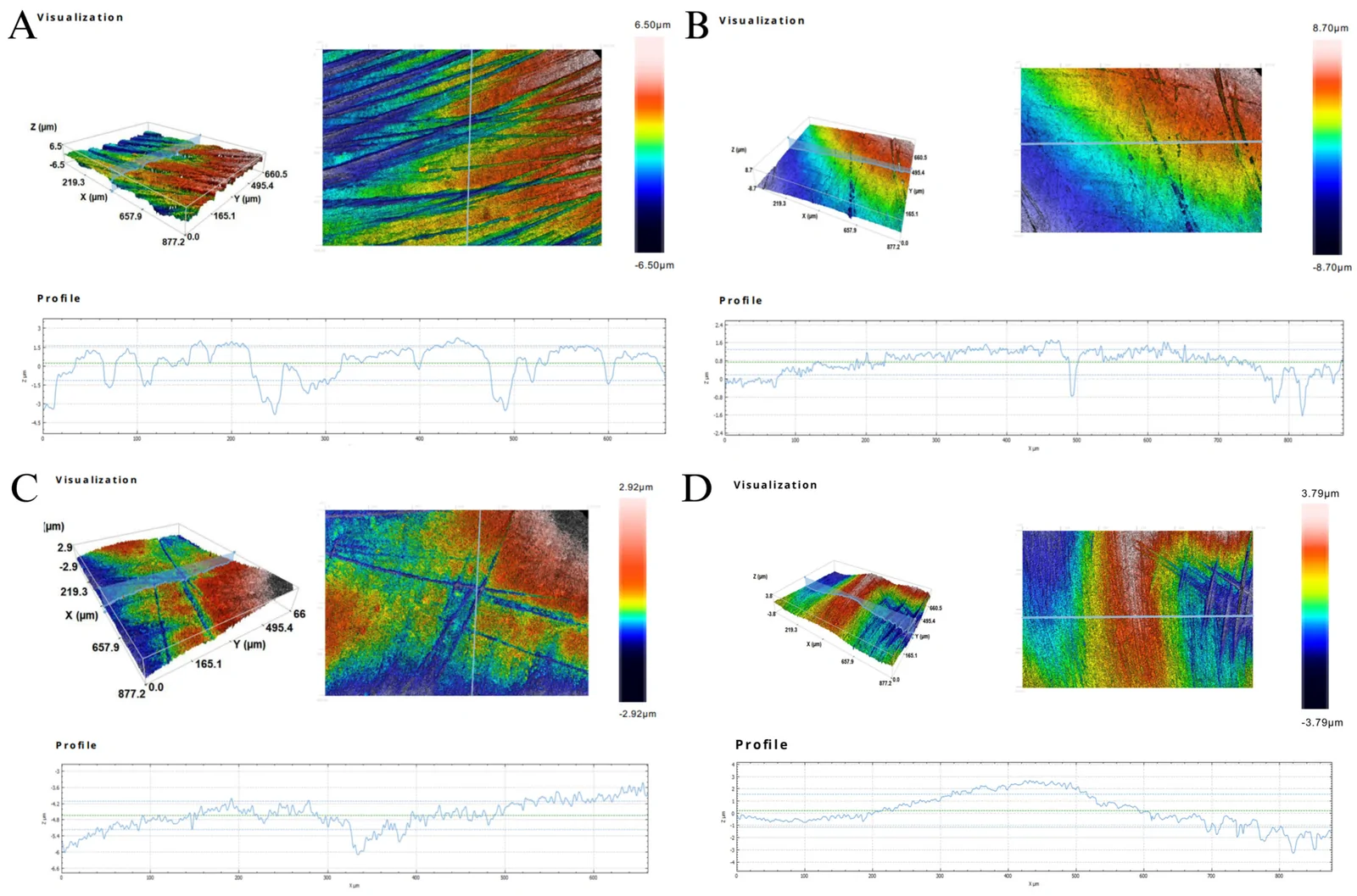
3D optical profilometry images of the NHC group: (**A**) control; (**B**) after thermocycling; (**C**) after Zaditen^®^ immersion; (**D**) after Zyrtec^®^ immersion. Profile graph represents the surface-height variation (Z, µm) along this measurement path (X, µm).

Descriptive evaluation of surface topography was performed using 3D optical profilometry and SEM. The 3D surface parameters obtained from the prospectively selected representative specimens are presented in Table 4; these values are descriptive and should not be interpreted as group means or quantitative group-level evidence. Representative SEM images obtained at ×500, ×5000, and ×10,000 magnifications are presented in Figure 1, Figure 2 and Figure 3, and the corresponding 3D surface reconstructions are shown in Figure 4, Figure 5 and Figure 6.

At baseline, the coated GH material exhibited a smooth, homogeneous, and compact surface without visible cracks or voids. Aging produced only mild surface irregularities, and the morphology after Zaditen^®^ exposure was comparable to that of the aged control. In contrast, Zyrtec^®^ exposure resulted in broad, deep, and localized erosive defects. NHC exhibited a granular and more heterogeneous baseline morphology, with increased roughness after aging. Zaditen^®^ exposure produced changes similar to aging alone, whereas Zyrtec^®^ exposure resulted in a more erosive surface, characterized by more prominent filler-particle protrusion. PMC showed the greatest aging-related surface deterioration. Although both syrups were associated with surface degradation, the extensive damage observed in the aged PMC control precluded a clear attribution of additional deterioration to either syrup.

In the GH group, control specimens exhibited an average roughness (Sa) of 12.42 µm, a maximum peak-to-valley height (Sz) of 63.12 µm, and a kurtosis value (Sku) of 2.04 (Table 4). Following Zaditen^®^ exposure, the parameters were recorded as Sa = 8.52 µm, Sz = 46.69 µm, and Sku = 2.14. Following exposure to Zyrtec^®^, the parameters changed to Sa = 4.23 µm, Sz = 19.35 µm, and Sku = 1.82 (Table 4, Figure 4). For the NHC material, control specimens showed Sa = 1.77 µm, Sz = 13.55 µm, and Sku = 2.55. After exposure to Zaditen^®^, the areal roughness was recorded as Sa = 0.69 µm, accompanied by a kurtosis value of Sku = 5.62 and Sz = 7.43 µm. Following immersion in Zyrtec^®^, the specimens showed Sa = 2.81 µm, Sz = 17.76 µm, and Sku = 2.58 (Table 4, Figure 6). For the PMC material, control specimens exhibited Sa = 4.17 µm, Sz = 25.36 µm, and Sku = 2.00. Zaditen^®^ exposure resulted in Sa = 1.71 µm, Sz = 20.59 µm, and Sku = 3.93. Following Zyrtec^®^ exposure, the parameters changed to Sa = 0.47 µm, Sz = 4.56 µm, and Sku = 5.18 (Table 4, Figure 5).

### 3.3. Color Stability (ΔE_00_) Evaluation

The mean total color-change values (ΔE_00_) and standard deviations are presented in Table 5. The three-way mixed-design ANOVA showed significant effects of time, material, immersion solution, and their interactions on color change (all *p* < 0.001). Statistical significance and clinical acceptability were interpreted separately, with ΔE_00_ = 1.80 used as the prespecified primary acceptability threshold [19].

GH showed the highest overall ΔE_3_ values among the tested materials. The highest mean ΔE_3_ value in the GH group was observed in the distilled-water control (6.50 ± 0.73), followed by the Zaditen^®^ group (5.04 ± 0.90). Both values exceeded the prespecified acceptability threshold of 1.80. Importantly, ΔE_3_ represents the cumulative color change between baseline and the final measurement and therefore includes changes occurring after thermocycling and subsequent solution exposure. Accordingly, the high final ΔE_3_ values in the GH groups should not be attributed solely to antihistamine exposure.

NHC and PMC generally showed lower ΔE_3_ values than GH. For NHC, the mean ΔE_3_ value was 2.73 ± 0.51 following Zaditen^®^ exposure and 4.46 ± 0.82 following Zyrtec^®^ exposure. For PMC, mean ΔE_3_ values ranged from 2.54 to 3.06 across the tested syrup groups. Although these values were lower than those observed for GH, all exceeded the prespecified acceptability threshold of 1.80. Therefore, these findings indicate threshold exceedance under the standardized experimental conditions of this in vitro study but should not be interpreted as direct evidence of clinically unacceptable color change or restoration failure.

## 4. Discussion

The aesthetic performance and surface integrity of pediatric restorative materials may be affected by variations in the oral chemical environment. Although the effects of dietary acids on dental materials are well documented, repeated exposure to pediatric oral liquid medications (POLMs), particularly antihistamine syrups prescribed for long-term use, represents an additional chemical challenge that warrants investigation [20,21]. The principal finding of this multimodal in vitro study was that changes in surface roughness, representative surface morphology, and color differed according to material, solution, and experimental stage. Importantly, a substantial proportion of the changes observed in GH occurred following thermocycling, before antihistamine exposure; therefore, its final response cannot be attributed solely to the tested syrups. The null hypotheses were rejected because significant effects of the investigated experimental factors were identified for surface roughness and color change. The large effect sizes observed for time and its interactions with material (η^2^p = 0.44–0.76) indicate that the magnitude of surface and color change over the experimental period was substantial and not merely statistically detectable; in contrast, the comparatively smaller effect size for the time × solution interaction on Ra (η^2^p = 0.18) suggests that the choice of antihistamine syrup contributed a more modest, though still significant, portion of the observed roughness change.

An important methodological consideration arising from the present study concerns the complementary assessment of surface alterations. Previous investigations of pediatric syrup exposure have relied predominantly on conventional two-dimensional roughness parameters such as Ra [22,23]. Although Ra provides quantitative group-level information on linear surface roughness, it may not fully characterize localized peaks, valleys, or heterogeneous surface features. Therefore, 3D optical profilometry was additionally used in accordance with ISO 25178-2 to provide descriptive areal information about the topography of prospectively selected representative specimens [15,24]. In the selected NHC specimen exposed to ketotifen and the PMC specimen exposed to cetirizine, relatively low Sa values were accompanied by higher Sku values, indicating that average areal roughness and the distribution of localized surface features may not change in parallel. Together with the corresponding SEM images, these observations may be compatible with localized matrix alteration and more prominent particle-like features [25]. However, because the 3D parameters were obtained from a single representative specimen per subgroup, they should not be interpreted as quantitative group-level evidence or as confirmation of a specific degradation mechanism. Thus, 3D optical profilometry and SEM provided complementary descriptive information that could not be captured by Ra alone, while the quantitative interpretation of surface roughness remained based primarily on the group-level Ra measurements. Because no compositional or chemical analyses (such as EDS or FTIR) were performed, these proposed mechanisms remain interpretations that require verification in future research.

The quantitative Ra findings may also be considered in relation to the commonly cited plaque-retention threshold of 0.20 µm proposed by Bollen et al. [9]. Surface roughness above this reference value has been associated with increased plaque accumulation in previous studies [26,27]. In the present study, the baseline Ra values of NHC were close to this threshold, whereas final Ra_t2 values following the complete experimental protocol were approximately 0.29–0.31 µm in the antihistamine groups. Higher final values were observed for PMC and GH, reaching approximately 0.42 µm and 1.01 µm, respectively. However, these final values reflect the cumulative effects of thermocycling and subsequent solution exposure and should not be attributed solely to the antihistamine syrups. Moreover, the 0.20 µm value was used only as a contextual reference rather than a definitive biological safety threshold. Because bacterial adhesion, biofilm formation, and secondary caries were not evaluated, the clinical consequences of threshold exceedance cannot be determined from the present findings. Although the composition and physical properties of pediatric liquid medications may influence their interaction with restorative surfaces, viscosity, titratable acidity, buffering capacity, and sugar content were not measured in this study. Therefore, their specific contributions to the observed changes remain uncertain.

The magnitude of the observed surface changes differed among the tested restorative systems. NHC generally showed lower absolute Ra values and smaller within-material changes than PMC and GH under the present experimental conditions. This observation is broadly consistent with previous studies reporting that some resin composites maintained lower surface roughness than other restorative material classes following exposure to pediatric liquid medications [7,13,28]. The response of NHC may be related to characteristics of its resin matrix, filler loading, and filler distribution, which can influence material behavior under hydrothermal and chemical exposure [23,29,30]. However, these compositional factors were not evaluated independently in the present study. Furthermore, because GH received a proprietary resin coating without mechanical polishing, whereas NHC and PMC were mechanically polished, differences in absolute Ra values cannot be attributed solely to material composition. Accordingly, greater emphasis should be placed on changes within each material relative to baseline rather than on a definitive between-material degradation hierarchy.

Within the resin-based materials, the response of the nanohybrid bulk-fill composite warrants specific consideration. Bulk-fill composites may not respond uniformly to hydrothermal and chemical exposure because their surface behavior can be influenced by resin-matrix composition, filler characteristics, and finishing procedures. In the present study, NHC showed no significant change in linear roughness following thermocycling alone, whereas Ra increased significantly from Ra_t1 to Ra_t2 following exposure to both antihistamine syrups. In the representative NHC specimen exposed to ketotifen, a relatively low Sa value was accompanied by a higher Sku value and localized particle-like surface features. These descriptive observations may be compatible with localized matrix alteration and increased filler prominence but do not confirm selective matrix erosion. The representative cetirizine-exposed specimen exhibited a comparatively more uniform pattern of surface irregularity. Because 3D profilometry and SEM were performed on prospectively selected representative specimens, these morphological observations cannot be generalized as quantitative group-level findings. Overall, NHC showed comparatively limited Ra changes under the present experimental conditions, although its surface was not unaffected by medication exposure. Similar variation among bulk-fill composites has been reported following thermocycling and acidic exposure, suggesting that post-aging roughness may be influenced by resin composition, filler characteristics, polishing procedures, and profilometric measurement conditions [17,24,31,32,33].

The roughness and representative topography findings for GH require cautious interpretation. Previous studies have suggested that nano-filled resin coatings may improve the surface hardness and wear resistance of glass-hybrid restorative systems under short-term or predominantly mechanical testing conditions [34,35]. In the present study, GH showed the highest final absolute Ra values, reaching 1.01 µm under the complete experimental protocol. However, a substantial proportion of the surface alteration was already observed following thermocycling, before antihistamine exposure. The representative 3D and SEM images showed localized layer separation, interfacial microgaps, and heterogeneous surface features that may be compatible with disruption of the resin coating and possible exposure of the underlying glass-hybrid material. Nevertheless, coating loss and exposure of the underlying core were not directly measured and therefore cannot be regarded as confirmed degradation mechanisms. Subsequent medication exposure was associated with additional changes, but these occurred on an already aged surface and should not be interpreted as the sole cause of the final GH response. Furthermore, because GH received a resin coating without mechanical polishing, whereas PMC and NHC were mechanically polished, comparisons of absolute Ra values among materials should be interpreted cautiously.

The changes observed in the GH system cannot be attributed solely to the chemical characteristics of the antihistamine syrups. Substantial color and surface changes were already evident following thermocycling, before medication exposure, and further changes were observed in the distilled-water control after completion of the experimental protocol. These findings indicate that hydrothermal aging contributed considerably to the final response of GH independently of antihistamine exposure. Previous studies have reported that the nano-filled resin coating of Equia Forte HT may undergo water sorption and hydrolytic alteration over time [35]. The present findings are consistent with the possibility that hydrothermal aging affected the coated GH surface; however, water sorption, solubility, coating thickness, and coating integrity were not directly measured. Similarly, although the representative surface images showed features potentially compatible with coating disruption, exposure of the underlying glass-hybrid core and subsequent pigment penetration were not directly demonstrated. Therefore, these mechanisms should be regarded as possible explanations rather than confirmed causes of the observed surface and color changes.

PMC showed intermediate absolute Ra values under the present experimental conditions. The representative SEM and 3D profilometry images exhibited localized matrix-like defects and prominent particle-like surface features, which may be compatible with matrix alteration and increased exposure of strontium–aluminum–fluorosilicate fillers. Similar morphological features have been reported for compomers following acidic and amoxicillin-syrup exposure [29,30]. However, because compositional changes were not directly analyzed and the morphological evaluations were based on prospectively selected representative specimens, matrix dissolution and filler exposure cannot be confirmed.

This study has several limitations. A major methodological limitation of this study lies in the differing surface preparation protocols between the materials. In accordance with the manufacturer’s specific clinical instructions to protect and maintain the Equia Forte Coat, the GH specimens did not receive any mechanical polishing, whereas the NHC and PMC specimens underwent standard mechanical polishing. While this approach reproduces the realistic clinical application of these materials, the lack of polishing in the GH group introduces a potential confounding factor when interpreting relative topographical changes. Secondly, the acidic exposure protocol simulated only the chemical dimension of the intraoral environment; mechanical wear and abrasive toothbrushing, which are known to act synergistically with acid challenges in accelerating degradation [15,22,36] were not incorporated. Thirdly, although 3D topographic profiling and SEM imaging were performed, subsurface microhardness and elemental EDS analyses were not included; furthermore, the quantitative 3D parameters were obtained from single representative specimens and must be interpreted as qualitative topographical trends rather than quantitative group means. Moreover, the immersion regimen—while adapted from a validated antihistamine-exposure model [17] is an accelerated in vitro model that represents a static, uninterrupted chemical challenge. Under real intraoral conditions, the liquid formulation is rapidly swallowed, diluted by saliva, and cleared dynamically. Therefore, the 5 min static immersion twice daily may overestimate the actual chemical exposure experienced in vivo. And lastly, the physicochemical characterization of the tested syrups was restricted to baseline pH measurements. Parameters such as titratable acidity, salivary buffering capacity, and formulation viscosity—which heavily govern the rate and depth of acidic erosion and matrix dissolution—were not quantified. These limitations must be considered when translating these laboratory findings to clinical outcomes. Long-term in vivo clinical tracking of pediatric patients receiving antihistamine therapy is warranted to consolidate the present findings.

## 5. Conclusions

Chronic exposure to ketotifen and cetirizine syrups was associated with changes in the surface topography and color stability of the three restorative materials tested, with material-dependent patterns rather than a uniform response to antihistamine exposure. The glass hybrid restorative system exhibited the greatest overall color change; however, a substantial proportion of this change was already observed following thermocycling, suggesting that the coated surface may have contributed to its color instability rather than attributing the observed changes solely to antihistamine exposure. The polyacid-modified composite and nanohybrid composite showed lower ΔE_00_ values than the glass hybrid system, although mean values in all groups exceeded the prespecified ΔE_00_ acceptability threshold of 1.80. However, because only a single product from each category was evaluated, these laboratory findings cannot establish a general superiority of resin-based materials as a class or support definitive clinical guidelines. Further long-term clinical trials are required to establish generalizable clinical recommendations for material selection in children receiving long-term antihistamines.

## Figures and Tables

**Table 1:** Syrups used in the study, their drug types, manufacturers, compositions, and pH values.

Medicine Name	Drug Type	Manufacturer	Composition	pH Values
Zaditen^®^	H1-antihistamine (ketotifen hydrogen fumarate 1.375 mg/5 mL)	Novartis Pharma AG, Basel, Switzerland	Ketotifen hydrogen fumarate; anhydrous citric acid; anhydrous disodium hydrogen phosphate; ethanol 94%; sucrose; sorbitol; strawberry flavor; purified water.	5.07
Zyrtec^®^	H1-antihistamine (cetirizine dihydrochloride 1 mg/mL)	UCB Pharma A.Ş., Istanbul, Türkiye	Cetirizine dihydrochloride; sorbitol solution (70%); glycerol; propylene glycol; sodium saccharin; methylparaben; propylparaben; banana flavor; sodium acetate; glacial acetic acid; purified water.	4.75

H1: histamine receptor.

**Table 2:** Materials and compositions of the restorative materials used in the study.

Material	Company	Category	Composition
Equia Forte HT	GC America, Alsip, IL, USA	Glass Hybrid Restorative System	Powder: Fluoroaluminosilicate glass, polyacrylic acid powder, iron oxide Liquid: Polybasic carboxylic acid, water
Equia Forte Coat	GC America, Alsip, IL, USA	Glass Hybrid Restorative System	Urethane methacrylate, methyl methacrylate, camphorquinone, colloidal silica, phosphoric ester monomer
Dyract Xp	Dentsply Sirona, Konstanz, Germany	Polyacid-Modified Composite Resin	Organic matrix: UDMA, Bis-GMA and TEGDMA; Filler: Glass ionomer–based filler particles (strontium–aluminum–fluorosilicate); camphorquinone.
Kulzer Bulk One	Kulzer, Hanau, Germany	Nanohybrid Resin Composite	Organic matrix: UDMA, EBADMA; barium aluminum boro-fluorosilicate glass; ytterbium fluoride; silica dioxide; titanium dioxide. Filler: Barium glass and silica-based inorganic fillers

UDMA: Urethane Dimethacrylate, Bis-GMA: Bisphenol A Glycidyl Methacrylate, TEGDMA: Triethylene Glycol Dimethacrylate, EBADMA: Ethoxylated Bisphenol A Dimethacrylate.

**Table 3:** Distribution of surface roughness (Ra, µm; mean ± SD) by material, solution, and time point (*n* = 10 per subgroup).

		NHC		GH	PMC	
		Mean ± SD		Mean ± SD	Mean ± SD	
Ra_t0	Control	0.27 ± 0.02		0.73 ± 0.11	0.36 ± 0.11	
	Zaditen^®^	0.23 ± 0.04		0.78 ± 0.15	0.35 ± 0.09	
	Zyrtec^®^	0.24 ± 0.05		0.73 ± 0.14	0.35 ± 0.07	
Ra_t1	Control	0.27 ± 0.03		0.79 ± 0.18	0.37 ± 0.12	
	Zaditen^®^	0.23 ± 0.03		0.79 ± 0.16	0.36 ± 0.09	
	Zyrtec^®^	0.26 ± 0.04		0.77 ± 0.13	0.36 ± 0.07	
Ra_t2	Control	0.28 ± 0.01		0.84 ± 0.16	0.42 ± 0.13	
	Zaditen^®^	0.29 ± 0.04		1.01 ± 0.10	0.41 ± 0.10	
	Zyrtec^®^	0.31 ± 0.04		0.96 ± 0.10	0.42 ± 0.06	
Source		Type III Sum of Squares	df	Mean Square	F	*p*
Time		0.439	2	0.219	137.807	<0.001 *
Time × Material		0.182	4	0.045	28.588	<0.001 *
Time × Solution		0.050	4	0.012	7.842	<0.001 *
Time × Material × Solution		0.036	8	0.005	2.837	0.006 *
Error (Time)		0.229	144	0.002		

Ra_t0: t0–t1 (effect of thermocycling); Ra_t1: t1–t2 (effect of solution exposure); Ra_t2: t0–t2 (overall change in surface roughness). NHC: Nanohybrid Resin Composite; GH: Glass Hybrid Restorative System; PMC: Polyacid-Modified Composite Resin. Statistical analysis was performed using repeated-measures ANOVA followed by the Bonferroni post hoc test. *p* < 0.05 was considered statistically significant. Mauchly’s test indicated that the assumption of sphericity was not violated for the within-subject factor time, χ^2^(2) = 4.33, *p* = 0.115; therefore, sphericity-assumed F values are reported. Partial eta-squared (η^2^p) with 95% confidence intervals: time, η^2^p = 0.657 (95% CI: 0.565–0.717); time × material, η^2^p = 0.443 (0.311–0.527); time × solution, η^2^p = 0.179 (0.063–0.270); time × material × solution, η^2^p = 0.136 (0.014–0.198). * Statistically significant difference (*p* < 0.05).

**Table 4:** ISO 25178-2 surface topography parameters of representative specimens from each experimental group at t2.

Material	Solution Group	Sa (µm)	Sz (µm)	Sp (µm)	Sv (µm)	Sku
NHC	Control	1.7711	13.550	6.2903	7.2597	2.5520
NHC	Zaditen^®^	0.6891	7.4323	4.6641	2.7682	5.6186
NHC	Zyrtec^®^	2.8100	17.764	6.0785	11.686	2.5797
GH	Control	12.419	63.120	33.021	30.099	2.0473
GH	Zaditen^®^	8.5167	46.690	23.602	23.088	2.1361
GH	Zyrtec^®^	4.2275	19.348	9.5719	9.7757	1.8188
PMC	Control	4.1654	25.360	12.372	12.989	1.9990
PMC	Zaditen^®^	1.7133	20.585	10.402	10.184	3.9345
PMC	Zyrtec^®^	0.4717	4.5583	1.3135	3.2447	5.1769

Sa: Arithmetical mean height of the surface; Sz: Maximum height of surface; Sp: Maximum peak height; Sv: Maximum valley depth; Sku: Kurtosis of the topography (a measure of the sharpness of the surface profile). NHC: Nanohybrid Resin Composite; GH: Glass Hybrid Restorative System; PMC: Polyacid-Modified Composite Resin. Note: Values were obtained from a single, prospectively and randomly selected specimen from each subgroup. Specimen selection was performed before the experimental procedures and was not based on surface roughness or morphological findings. These measurements are presented solely to descriptively illustrate the surface topography of the selected specimens and should not be interpreted as quantitative group-level evidence or group means.

**Table 5:** Distribution of color change (ΔE_00_; mean ± SD) by material, solution, and time interval (*n* = 10 per subgroup).

		NHC		GH	PMC	
		Mean ± SD		Mean ± SD	Mean ± SD	
ΔE1	Control	1.86 ± 0.15		6.34 ± 1.19	2.16 ± 0.13	
	Zaditen^®^	3.08 ± 0.82		4.59 ± 0.44	2.28 ± 0.19	
	Zyrtec^®^	1.94 ± 0.26		2.69 ± 0.65	2.06 ± 0.46	
ΔE2	Control	3.62 ± 0.54		1.64 ± 0.37	2.87 ± 0.35	
	Zaditen^®^	3.72 ± 0.44		2.09 ± 1.07	2.88 ± 0.41	
	Zyrtec^®^	3.81 ± 0.55		1.41 ± 0.37	2.84 ± 0.28	
ΔE3	Control	3.57 ± 0.50		6.50 ± 0.73	3.06 ± 0.31	
	Zaditen^®^	2.73 ± 0.51		5.04 ± 0.90	3.02 ± 0.75	
	Zyrtec^®^	4.46 ± 0.82		2.90 ± 0.65	2.54 ± 0.58	
Source		Type III Sum of Squares	df	Mean Square	F	*p*
Time		42.466	2	21.233	62.132	<0.001 *
Time × Material		153.332	4	38.333	112.172	<0.001 *
Time × Solution		15.490	4	3.872	11.332	<0.001 *
Time × Material × Solution		38.677	8	4.835	14.147	<0.001 *
Error (Time)		49.210	144	0.342		

ΔE_1_: Color change between baseline (t0) and post-thermocycling (t1); ΔE_2_: Color change between post-thermocycling (t1) and post-immersion (t2); ΔE_3_: Overall color change between baseline (t0) and post-immersion (t2). NHC: Nanohybrid Resin Composite; GH: Glass Hybrid Restorative System; PMC: Polyacid-Modified Composite Resin. Statistical analysis was performed using a three-way mixed design ANOVA followed by the Bonferroni post hoc test. *p* < 0.05 was considered statistically significant. Mauchly’s test indicated that the assumption of sphericity was not violated for the within-subject factor time, χ^2^(2) = 0.42, *p* = 0.812; therefore, sphericity-assumed F values are reported. Partial eta-squared (η^2^p) with 95% confidence intervals: time, η^2^p = 0.463 (95% CI: 0.342–0.551); time × material, η^2^p = 0.757 (0.684–0.798); time × solution, η^2^p = 0.239 (0.112–0.334); time × material × solution, η^2^p = 0.440 (0.291–0.511). * Denotes statistically significant interactions.

## Data Availability

The data that support the findings of this study are not publicly available due to privacy restrictions but are available from the corresponding author upon reasonable request.
